# Thin MAPb_0.5_Sn_0.5_I_3_ Perovskite Single Crystals for Sensitive Infrared Light Detection

**DOI:** 10.3389/fchem.2021.821699

**Published:** 2022-01-07

**Authors:** Jinming Wu, Yongqiang Zhang, Shuang Yang, Zhaolai Chen, Wei Zhu

**Affiliations:** ^1^ State Key Laboratory of Crystal Materials, Institute of Crystal Materials, Shandong University, Jinan, China; ^2^ Institute of Radiation Medicine, Shandong Academy of Medical Sciences, Shandong First Medical University, Jinan, China; ^3^ Sunzhou Research Institute, Shandong University, Suzhou, China; ^4^ LinYi Ecological Environmental Bureau, LinYi, China

**Keywords:** perovskite, single crystal, near-infrared detection, low toxicity, operational stability

## Abstract

Metal halide perovskite single-crystal detectors have attracted increasing attention due to the advantages of low noise, high sensitivity, and fast response. However, the narrow photoresponse range of widely investigated lead-based perovskite single crystals limit their application in near-infrared (NIR) detection. In this work, tin (Sn) is incorporated into methylammonium lead iodide (MAPbI_3_) single crystals to extend the absorption range to around 950 nm. Using a space-confined strategy, MAPb_0.5_Sn_0.5_I_3_ single-crystal thin films with a thickness of 15 μm is obtained, which is applied for sensitive NIR detection. The as-fabricated detectors show a responsivity of 0.514 A/W and a specific detectivity of 1.4974×10^11^ cmHz^1/2^/W under 905 nm light illumination and –1V. Moreover, the NIR detectors exhibit good operational stability (∼30000 s), which can be attributed to the low trap density and good stability of perovskite single crystals. This work demonstrates an effective way for sensitive NIR detection.

## Introduction

Metal halide perovskite with an ABX_3_ structure shows promising potential for various applications, such as detectors and solar cells (Xiao et al., 2014), light-emitting diodes, lasers, and field-effect transistors, due to long carrier diffusion length, tunable optical bandgaps, flexibility, low cost and easy fabrication (Ju et al., 2019). Recently, the power conversion efficiency of perovskite solar cells has reached 25.5% (Best Research-Cell Effici, 2021), which sets a new efficiency benchmark for solution-processed solar cells. Perovskite photodetectors also show excellent performances such as fast response speed, high detectivity, low noise, and large linear dynamic range (Li et al., 2020).

Up to now, the most widely studied perovskite detectors are based on polycrystalline films, which included grain boundary, small grain sizes, and pinhole and low surface coverage (Wang and Kim, 2017; Yang et al., 2017; Li et al., 2019; Zhao et al., 2020). A slow crystallization process is believed to provide a universal strategy to improve crystal quality (Liu et al., 2018). Compared to polycrystalline thin films, higher crystallization quality and longer carrier diffusion length can be observed in single crystals (Huang et al., 2015; Liu et al., 2016). Additionally, the remarkable stability of single crystals could enhance the stability of optoelectronic devices (Zhang Y et al., 2018). For example, Bao et al. (2017) reported visible-light photodetectors based on MAPbI_3_ (iodine) and MAPbBr_3_ (bromine) single-crystal thin film with high photoresponsivity, low noise, and large linear dynamic range. Chen et al. (2019a) fabricated visible-blind UV (ultraviolet) photodetectors based on MAPbCl_3_ (chlorine) single-crystal thin film with 15-ns response time.

It should be noted that perovskite single crystals based on Pb show a typical optical bandgap (E_g_) > 1.5 eV, which limits the capture of photons in the NIR region. To obtain sufficient optical absorption in the NIR region, a new component and other sensitizers can be introduced into Pb (lead)-based perovskite. Among all the alternative elements for the toxic Pb element, Sn is a promising candidate for the extension of the absorption range. Zhu et al. (2018) reported photodetectors based on Sn–Pb mixed polycrystalline thin films with A-cation engineering, which achieved larger photocurrent and lower noise current between 340 and 1000 nm. You et al. reported stable Pb–Sn mixed low-bandgap perovskite solar cells with composition engineering (Chi et al., 2018). By adjusting the ratio of Pb^2+^ and Sn^2+^, the absorption range of mixed Pb–Sn perovskite can be extended into the NIR region, which is an effective and green strategy to achieve NIR response. Therefore, it is important for developing Pb–Sn mixed single-crystal thin films as a light absorption layer for NIR detectors.

In this work, MAPb_0.5_Sn_0.5_I_3_ single-crystal thin films with a thickness of 15 μm and high crystallization quality are grown by using the space-confined method. Partial substitution of Pb in MAPbI_3_ with Sn can reduce toxicity and lower the bandgap. The employment of MAPb_0.5_Sn_0.5_I_3_ single-crystal thin films in NIR detectors has been demonstrated with high detectivity and operational stability, which provides a less-toxic alternative material for sensitive NIR detection.

## Materials and Methods

### Materials

Chemicals and reagents: Methylcholine iodide (MAI) was synthesized from stannic iodide produced by Xi’an Polymer Light Technology. Gamma-butyrolactone (GBL, 99%) was purchased from Aladdin Reagent Ltd. Fullerene (C60), 2,9-dimethyl-4,7-diphenyl-1,10-phenathroline (BCP), and polytriaryl amine (PTAA) were purchased for Xi’an Polymer Light Technology.

Preparation of MAP_b0.5_Sn_0.5_I_3_ single-crystal thin film: indium tin oxide (ITO) substrates were washed in deionized water about 15 min by an ultrasonic cleaner (KS-3200E), then a surface treatment by ultraviolet–ozone (UVO) for 10 min was carried out. Next, the hydrophobic and conductive films were fabricated by a spin coating PTAA solution (0.2 wt% in chlorobenzene) at 3,000 rpms, and subsequently annealed at 100°C for 15 min.

The single-crystal thin film was grown by using the space-confined method with a hydrophobic interface. A drop of the prepared 1.7M MAPb_0.5_Sn_0.5_I_3_ precursor solution in GBL was inserted into two ITO covered by PTAA substrates. Then, substrates were placed on a hot plate with a temperature of 45°C for 10 min. Subsequently, the temperature was raised to 95°C to further promote the nucleation and growth of single-crystal thin films. Finally, the two substrates were carefully separated to get the single-crystal thin film on substrates with a blade.

Infrared Detector Device Fabrication:C60 (40 nm) and BCP (3 nm) were thermally evaporated at a rate of 0.2 Å/s to form the charge transport layer. Cu (80 nm) was thermally evaporated at a rate of 0.8 Å/s to form the electrode. Au (40 nm) was thermally evaporated at a rate of 0.6 Å/s to form the electrode.

### Characterizations

#### X-Ray Diffraction

Powder X-ray diffraction (PXRD) patterns were measured on a smartLab SE High Resolution Diffraction System with Cu Kα1 radiation (*λ* = 1.54186Å) in the range of 5–90°(2θ) with a single-crystal thin film of 2 × 1 × 0.02 mm^3^ in size.

#### Scanning Electron Microscope

The surface and cross-morphologies images were taken from a field emission (Phenom Pharos).

Steady-state absorption: Absorption spectra were determined using a U3500 Hitachi UV/Vis Spectrophotometer with the self-made mold of test.

#### Energy-Dispersive Spectrometer

The element components with a single-crystal thin film of 2 × 1 × 0.02 mm^3^ in size were measured by using a desktop field-emission scanning electron microscope energy spectrum all-in-one machine (Phenom Pharos).

#### Thermogravimetric Analysis and Differential Scanning Calorimetry Measurements

TGA and DSC curves were characterized by using a TGA/DSC analyzer (Setaram). The powder, which was collected using by a single-crystal thin film, was placed in an aluminum crucible and heated at a rate of 10 K/min from room temperature to 800°C under flowing nitrogen gas.

#### Device Characterizations

Device I-V characteristics were collected by using a Keithley 2400 analyzer. AM 1.5-G irradiation (100 mW/cm^2^) was produced by a xenon lamp-based solar simulator. NIR laser of 10 mW intensity was provided by the NIR light source (ZLM50AX905-16GD). A tunable power was carried out by the adjustment of Cu film thickness. P_inc_ = P/A, where A is the active area of the light spot, P_inc_ is the incident power density, and P is the incident power.

## Results and Discussion

**FIGURE 1:**
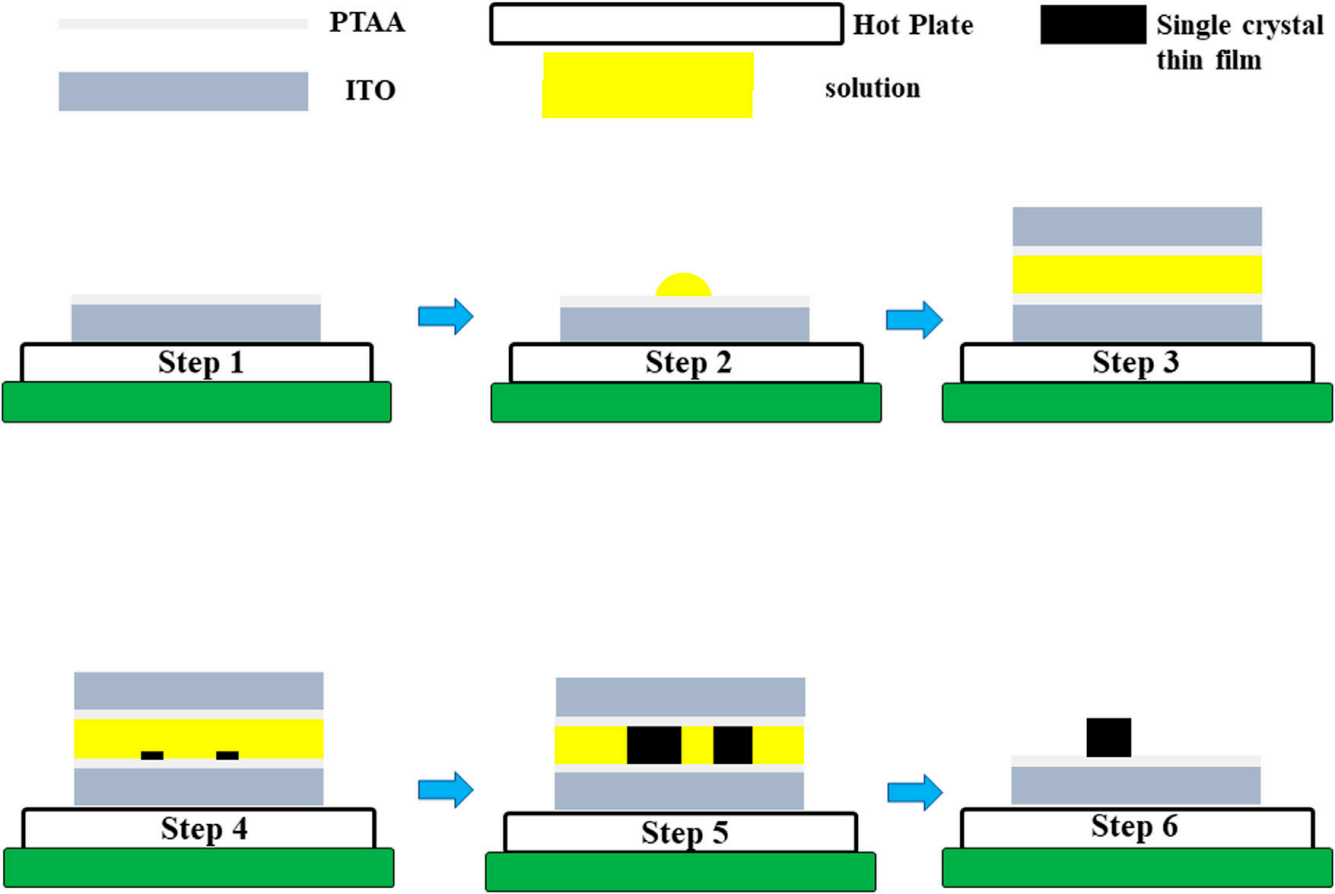
Schematic illustration of single-crystal thin film growth process by using the space-confined method.

The inverse temperature crystallization (ITC) method was applied for the growth of MAPb_0.5_Sn_0.5_I_3_ single crystals in which solubility decreases with increasing temperature (Saidaminov et al., 2015). To grow micrometer-thick single-crystal thin films, the precursor solution was inserted into two ITO substrates, and the crystal thickness was determined by the substrate gap (Figure 1).

**FIGURE 2:**
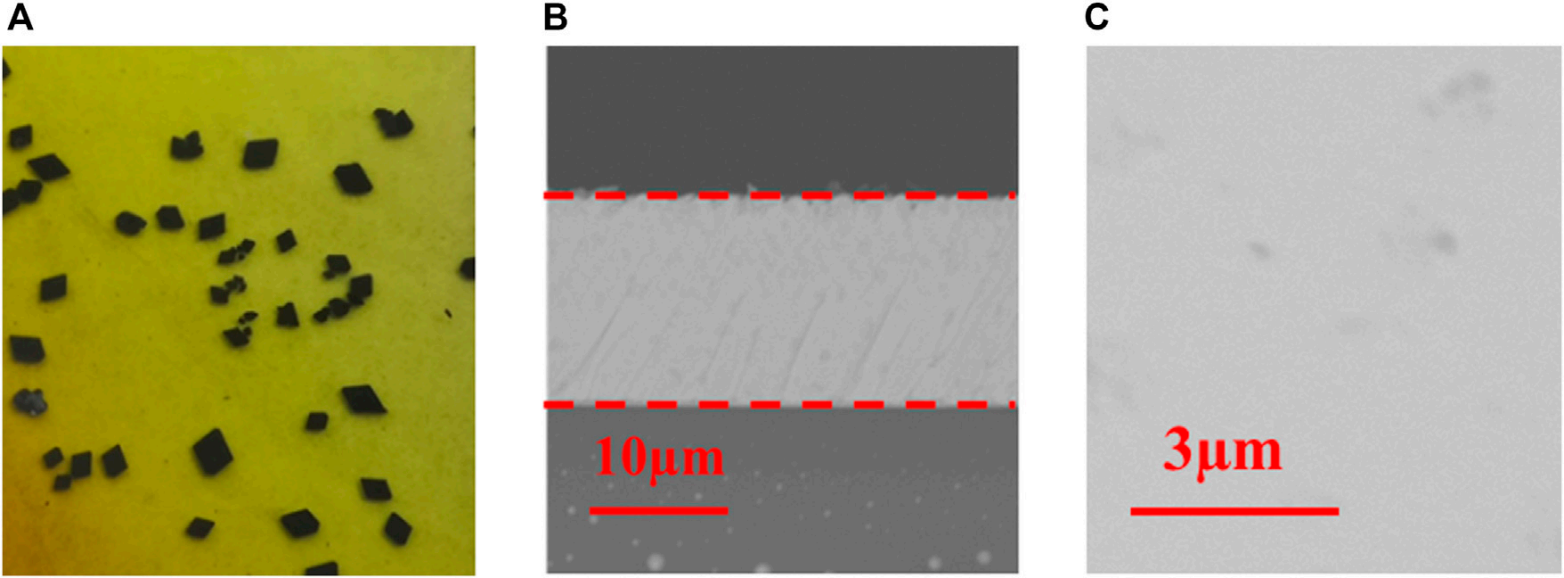
**(A)** Photograph, **(B)** cross-sectional SEM images, and **(C)** surface morphology SEM images of a MAPb_0.5_Sn_0.5_I_3_ single-crystal thin film.

Figure 2A shows the photographs of as-grown MAPb_0.5_Sn_0.5_I_3_ single-crystal thin films with a thickness of 15 μm. The crystal shape is hexagonal, which is similar to that of MAPbI_3_ single-crystal thin films. SEM is employed to study the surface thickness and morphology. It can be seen from the cross-sectional SEM image (Figure 2B) that the thickness of the single-crystal thin film is about 15 µm. The top-view SEM image (Figure 2C) indicates the absence of grain boundaries for a single-crystal thin film and the surface is very smooth.

**FIGURE 3:**
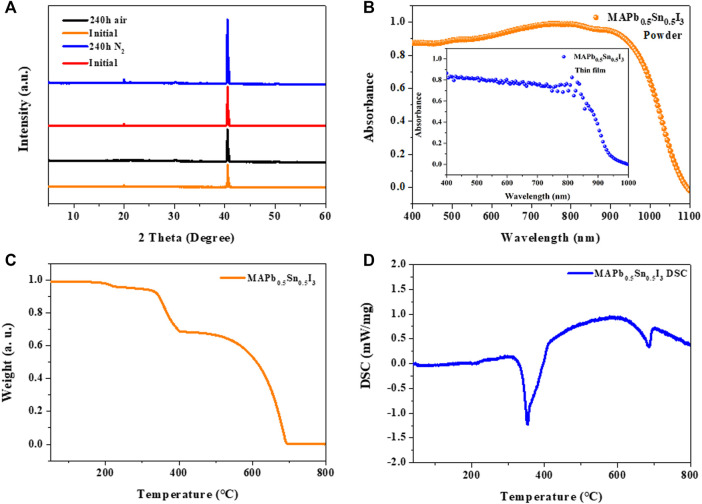
**(A)** XRD pattern of a MAPb_0.5_Sn_0.5_I_3_ single-crystal thin film. **(B)** UV-Vis-NIR absorption normalized spectra of thin film and UV-Vis diffuse reflectance of powder. **(C)** TGA and **(D)** DSC data for MAPb_0.5_Sn_0.5_I_3_.

In Figure 3A, the XRD patterns of MAPb_0.5_Sn_0.5_I_3_ single-crystal thin films mainly show two diffraction peaks at 20.03º and 40.51º, which can be assigned to (110) and (220) planes, indicating that the MAPb_0.5_Sn_0.5_I_3_ single-crystal thin films are grown along the [110] orientation. Furthermore, it can be noted that the XRD patterns of the MAPb_0.5_Sn_0.5_I_3_ single-crystal thin film show negligible change after storage in air and N_2_ atmosphere for 10 days, implying its good structure stability. As shown in Figure 3B, the absorption onset peak is 950 nm for MAPb_0.5_Sn_0.5_I_3_ single-crystal thin film, which corresponds to a suitable small bandgap of 1.3 eV. The conjunction of a glass substrate and ITO electrodes exhibits a fully transparent device for the incident light direction. To examine the thermal stability of MAPb0.5Sn0.5I3 single crystal thin film from the substrates and then measured by TGA and DSC (Figures 3C,D). The TGA curve of MAPb_0.5_Sn_0.5_I_3_ exhibits obvious mass loss after 170°C and comprises three-step decomposition process, including the evaporation of MAI, SnI_2_, and PbI_2_. In the DSC curve, a clear exothermal peak is shown at 350°C, corresponding to the melting point of MAPb_0.5_Sn_0.5_I_3_.

**FIGURE 4:**
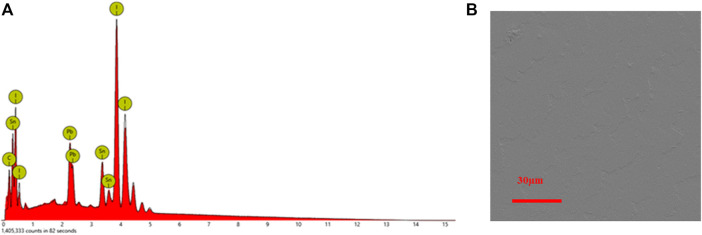
**(A)** Energy-dispersive X-ray spectroscopy of MAPb_0.5_Sn_0.5_I_3_ in **(B)** surface zone.

EDS measurement is adopted to analyze the chemical composition of the as-grown single-crystal thin films. According to Table 1 and Figure 4, we can verify that single-crystal thin films with Pb/Sn ratio of nearly 1:1 are synthesized by combining the ITC and space-confined methods. The aforementioned results confirm successful preparation of MAPb_0.5_Sn_0.5_I_3_ single-crystal thin films with good structure stability and absorption extended into the NIR region.

**TABLE 1:** SEM–EDS results of element components and ratio of a MAPb_0.5_Sn_0.5_I_3_ single-crystal thin film.

Element symbol	Weight/%	Atomic/%	Ratio
I	63.84	50.07	5.4962
C	3.67	30.43	3.34
Pb	21.62	10.39	1.1405
Sn	10.86	9.11	1

**FIGURE 5:**
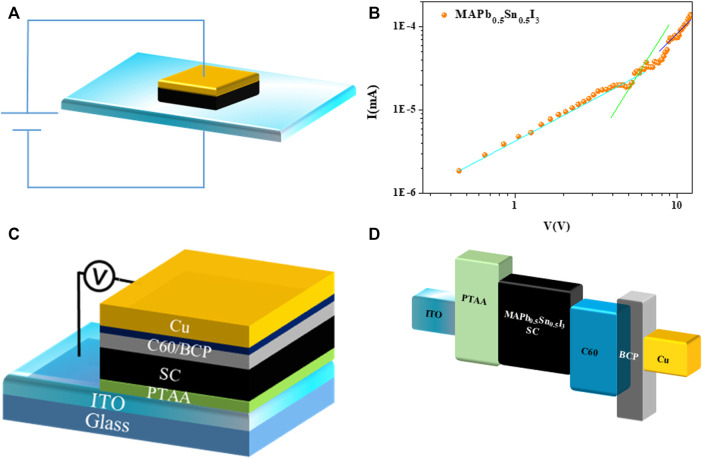
**(A)** Schematic device structure. **(B)** Dark current–voltage curves of hole-only devices based on a MAPb_0.5_Sn_0.5_I_3_ single-crystal thin film. **(C)** Schematic device structure and **(D)** energy band diagram of MAPb_0.5_Sn_0.5_I_3_.

Superior optoelectronic properties are required for high-performance photodetectors. To characterize the hole mobility and hole trap density of as-grown MAPb_0.5_Sn_0.5_I_3_ single-crystal thin films, hole-only devices with the structure of ITO/PTAA/single crystals/Au were fabricated, as is shown in Figure 5A. The hole mobility and trap density are calculated according to the following two equations:

$$J=\frac{9\varepsilon \varepsilon_{0}µV^{2}}{8d^{3}},$$ (1)

where *ε* is the relative dielectric constant, *ε*_*0*_ is the dielectric constant of free space, *d* is the film thickness, *V* is the bias, and *µ* is carrier mobility;

$$n_{trap}=\frac{2\varepsilon \varepsilon_{0}V_{TEL}}{qd^{2}},$$ (2)

where *V*_*TFL*_ is the voltage at which all the traps are filled, *e* is the elementary charge, and *n*_t_ is the hole trap density (Zhang Y et al., 2018; Kim et al., 2021). The hole mobility and trap density are calculated to be 0.001485 cm^2^V^−1^s^−1^ and 6.948×10^13^ cm^−3^ (Figure 5B), respectively. In contrast to the pure lead-based perovskite single crystals, the mobility is relatively low, while the trap density is relatively high, which should be due to the oxidation of Sn^2+^ to Sn^4+^. Future optimization can be focused on how to avoid the oxidation of Sn^2+^, such as introduction of reductants, which is under investigation in our laboratory.

To explore the application of MAPb_0.5_Sn_0.5_I_3_ single-crystal thin films in NIR detection, photodetectors were easily fabricated by depositing the electron transport layer and metal electrode, benefiting the substrate/hole transport layer (HTL)–integrated growth of single-crystal films (Chen et al., 2019b). As shown in Figure 5C, the device structure of ITO/PTAA/MAPb_0.5_Sn_0.5_I_3_ single-crystal thin film/C_60_/bathocuproine (BCP)/copper (Cu). The hydrophobic PTAA layer functions as both the hole transporting layer and non-wetting interface, which is beneficial for the improvement of crystallization and fabrication of the device (Bi et al., 2015). The BCP layer can enhance the surface smoothness of C_60_ and reduce the trapped electrons (Kavadiya et al., 2020; Ying et al., 2021). The energy diagram of the detector is shown in Figure 5D, exhibiting that the PTAA hole transport layer and C_60_/BCP electron transport layer can promote the charge extraction at zero bias (Meng et al., 2016).

**FIGURE 6:**
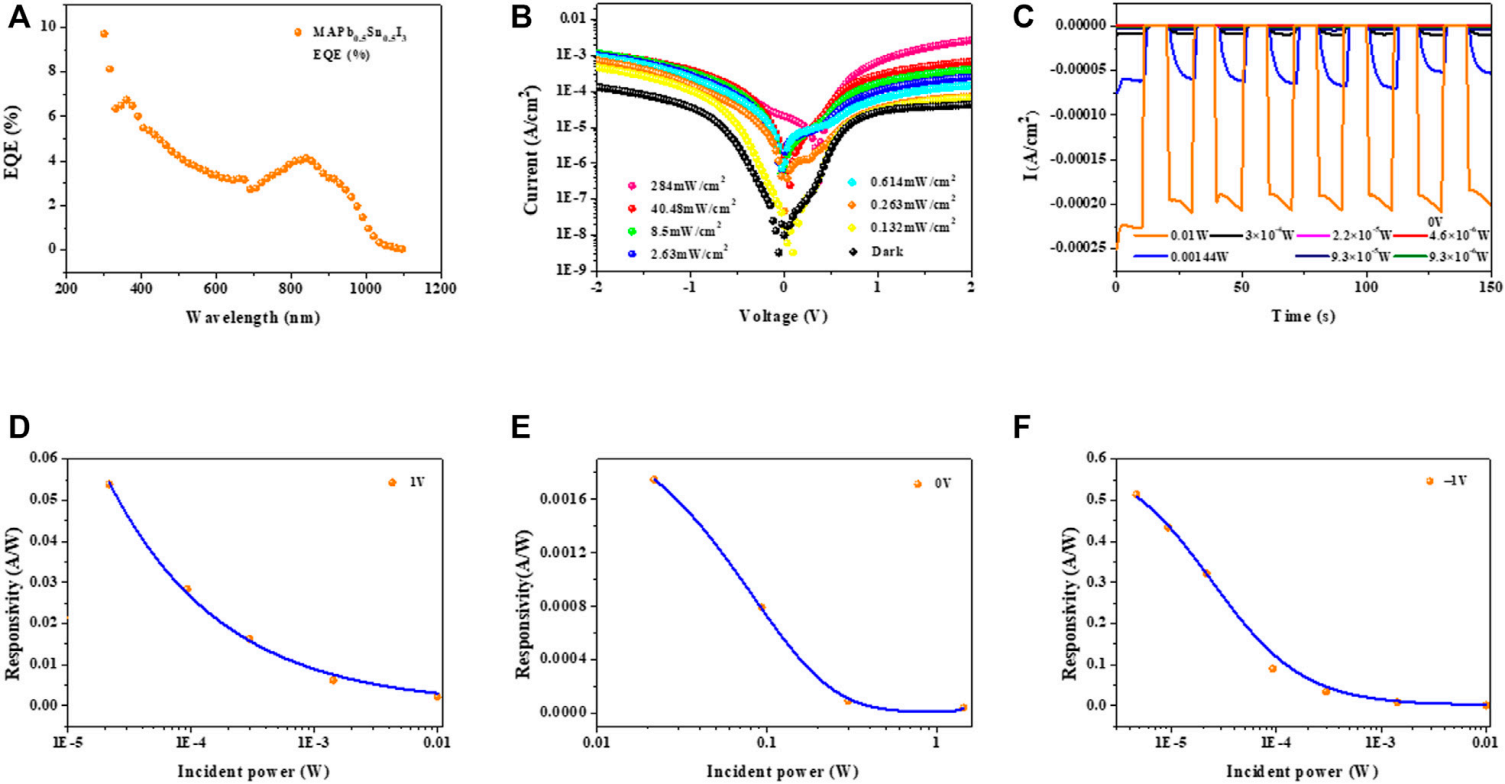
**(A)** EQE curves of MAPb_0.5_Sn_0.5_I_3_ single-crystal devices under zero bias. **(B)** Current–voltage curves of a MAPb_0.5_Sn_0.5_I_3_ single-crystal devices under 905 nm near-infrared light illumination and dark. A single-crystal thin film device based on MAPb_0.5_Sn_0.5_I_3_ response to 905 nm near-infrared light by turning the source on and off **(C)** at zero bias. Relationship between the photocurrent and the incident light intensity at 1V **(D)**, 0V **(E)**, and –1V **(F)**.

Effective carrier collection plays a significant role in responsivity of the detectors, which is evaluated by the external quantum efficiency (EQE) measurement under zero bias (Fang et al., 2015). The EQE is defined as the ratio between the number of photogenerated carriers and incident photons at a certain wavelength, which can be applied for evaluating the photoelectric conversion ability of photodetectors (Dong Q et al., 2015; Liu et al., 2019). As shown in Figure 6A, EQE values of 3% for MAPb_0.5_Sn_0.5_I_3_ single-crystal detectors at 905 nm are obtained under zero bias. The EQE values are consistent with the relative low mobility of MAPb_0.5_Sn_0.5_I_3_ single-crystal films.

To directly display the performance of NIR detectors, current density–voltage (J-V) measurements were carried out in the dark and illuminated with 905 nm light (Figure 6B). When the light intensity increases from 0.13 mW/cm^2^ to 284 mW/cm^2^, the photovoltage increases from 0.09 to 0.36 V. The existence of a photovoltage indicates the NIR photodetectors can work without external bias. Therefore, we mainly focus on the device performance under zero bias, which is so-called self-powered photodetectors. The responsivity (R) and detectivity (D*) are key parameters for evaluating the performances of photodetectors (Liu et al., 2018), which can be calculated according to the following equations:

$$R=\frac{\left|I_{photo}\right|}{P_{light}\times A}$$ (3)

$$D^{*}=\frac{R}{{\left(2qI_{\text{dark}}/A\right)}^{\frac{1}{2}}},$$ (4)

where *I*_*photo*_ and *P*_*light*_ are the photocurrent and incident optical power, *A* is the area of device, *q* is the charge value of an electron, and *I*_*dark*_ are the dark currents. In the time-dependent photocurrent curve, it can be noted that the on/off ratio was about 10^3^, at 284 mWcm^−2^ incident light intensity, demonstrating an obvious response for 905 nm at 0V bias (Figure 6B). When the incident light intensity was adjusted to 40.48 mWcm^−2^, the response current is strikingly measured to reduce from 2 × 10^−5^ Acm^−2^ to 1.5 × 10^−6^ Acm^−2^, for which the comparison can be attributed to the defects (Figures 6B,C). But the response current still shows the obvious difference until 4.65 × 10^−6^W (0.13161 mWcm^−2^) at an applied bias of 0V (Figure 6C). With all the aforementioned measurement device performances, we demonstrated a promising NIR detector at zero bias with the employment of MAPb_0.5_Sn_0.5_I_3_ single-crystal thin films. We further measured the detector performance by using a quantitative analysis for the device under zero bias and –1V and 1V, respectively. We further investigated the relationship between the responsivity and light intensity. Figures 6D–F exhibits the light density–dependent responsivity, and the result proves our MAPb_0.5_Sn_0.5_I_3_ based on the NIR detector showing apparent intensity-dependent characteristics, and a lower light intensity leads to a larger responsivity. Responsivity as a function of the light intensity is shown in Figure 6D with bias at 1V. The responsivity decreases at high incidence, and this mechanism can attribute to previous reports for electronic trap states at the perovskite–ITO interface (Muñoz et al., 1997; Konstantatos et al., 2006; Lhuillier et al., 2013; Dong R et al., 2015). The NIR photodetector exhibits a responsivity of 0.0539 A/W and a detectivity of 1.86×10^10^ cmHz^1/2^W^−1^ at 1 V bias. Under 0.6143 mWcm^−2^ incident light intensity of 905 nm, the highest *R* and D* are calculated to be 0.01746 A/W and 3.08×10^10^ cmHz^1/2^W^−1^ at zero bias (Figure 6E), respectively. In addition, at low power (4.65 × 10^−6^ W), responsivity is measured to be 0.514 A/W at –1V bias (Figure 6F). Based on the combination of information in the dark and the responsivity, we could calculate the detectivity about 1.497×10^11^ cmHz^1/2^W^−1^ at –1V bias.

**FIGURE 7:**
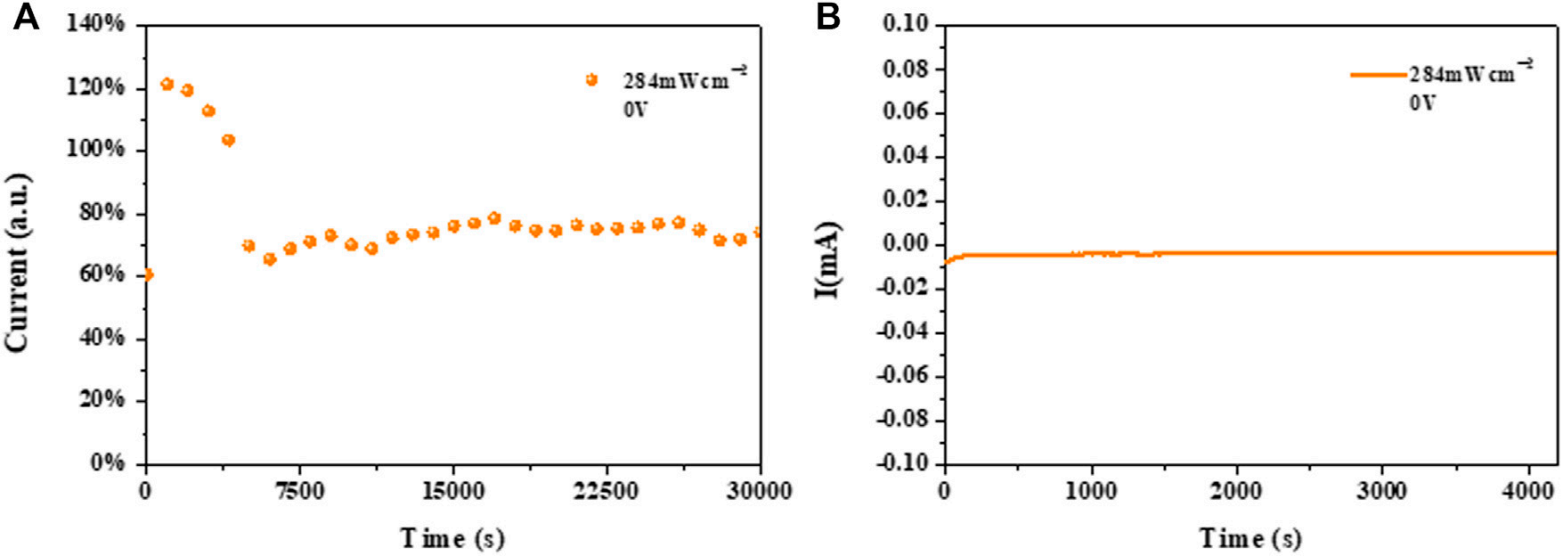
**(A)** Operational stability of a MAPb_0.5_Sn_0.5_I_3_ single-crystal device under 284 mW/cm^2^ 905 nm near-infrared light with continuous illumination at zero bias. **(B)** Response current stability of device based on a MAPb_0.5_Sn_0.5_I_3_ single crystal under 284 mW/cm^2^.

Finally, we further investigated the operational stability for the single-crystal thin films based on the NIR detector. The lifetime of detector is mainly determined by the operational stability (Lei et al., 2018; Wu et al., 2021). As shown in the Figure 7A, the device maintained average 70% operational performance, irradiated by 905 nm NIR light source with 284 mW/cm^2^ after continuous 30000 s in N_2_ atmosphere. Figure 7B shows that the detector showed a stable response current up to 4000 s at zero bias.

## Discussion

The oxidation of Sn^2+^ increased the deep traps for an obvious decrease in the photocurrent, which becomes a critical factor for the carrier transport (Cheng et al., 2020). The photocurrent declines distinctly when decreasing the light intensity, which was originated from capture of deep traps of the surface for carriers. The photogenerated carriers were captured by the main deep traps from the surface, and the interface of poor contact, ^[34]^ as a result, photocurrent exhibited the reduction in an order of magnitude or more.

## Conclusion

In summary, in this work, we demonstrate the first efficient NIR detector with MAPb_0.5_Sn_0.5_I_3_ single-crystal thin films grown by using the space-confined method. The mixed Pb–Sn element can enable a wider response range for NIR detection. In addition, the single-crystal thin films with reduced Pb contents exhibit less toxicity, and the partial existence of Pb can avoid severe oxidation of Sn^2+^ in pure Sn-based perovskites, leading to an enhanced operational stability of detectors. Notably, the MAPb_0.5_Sn_0.5_I_3_-based NIR detector shows obvious photoresponse at zero bias and a responsivity of 0.0016 A/W as well as a detectivity of 3.08×10^10^ cmHz^1/2^/W. We believe that the incorporation of Sn in Pb-based perovskite single-crystal thin films will provide an alternative route for achieving efficient NIR detectors.

## Data Availability

The original contributions presented in the study are included in the article/supplementary material; further inquiries can be directed to the corresponding authors.
